# Human continuous glucose monitors for measurement of glucose in dairy cows

**DOI:** 10.3168/jdsc.2021-0147

**Published:** 2021-11-18

**Authors:** M.K.H. Byrd, A.G. Arneson, D.R. Soffa, J.W. Stewart, M.L. Rhoads

**Affiliations:** Department of Animal and Poultry Sciences, Virginia Tech, Blacksburg 24061

## Abstract

If validated for use in dairy cattle, interstitial continuous glucose monitors (CGMs) could be easily implemented, informative tools for research, clinical, and perhaps even on-farm applications. To evaluate their efficacy, 2 experiments were conducted, during which lactating Holstein cows were fit with indwelling jugular catheters, as well as FreeStyle Libre (FSL; Abbott) and Dexcom G6 (DexCom Inc.) CGMs secured either behind their polls, lateral to their ears, or beneath their pin bones on their upper rear legs. During the first experiment, blood (measured with a handheld glucometer) and interstitial glucose measurements were collected from 13 cows every 4 h for 96 h. In the second experiment, the same measurements were collected from 8 cows every 15 min for 6 h. At the mid-point of the sampling period (3 h), cows received a bolus dose of dextrose to facilitate comparisons across a broad range of glucose concentrations. Results from both experiments determined that functional longevity of the sensors was greatest for those sensors secured near the ear. Likewise, interstitial measurements from the ear sensors were most closely correlated with blood glucose concentrations (r = 0.82 and r = 0.71 for FSL ear and Dexcom G6 ear, respectively). Unfortunately, accuracy calculated as absolute relative error was low, at 60.7% or less. As a result of the low accuracy, even though both ear sensors detected an increase in glucose concentrations following the bolus dose, neither produced results exactly matching blood glucose measurements. The results of this work indicate that the FSL and Dexcom G6 CGMs are not currently capable of replacing blood-based glucose measurements.

## Highlights

• Continuous glucose monitors of 2 brands were applied behind the poll near the ear or on the upper rear leg.
• Both ear sensors detected an increase in glucose following a bolus dose but neither exactly matched blood glucose measurements.
• Functional longevity of the sensors was greatest for those sensors secured near the ear, but accuracy was low.
• Neither of the sensors is capable of replacing blood-based glucose measurements in dairy cows.

## Graphical Abstract

**Figure:**
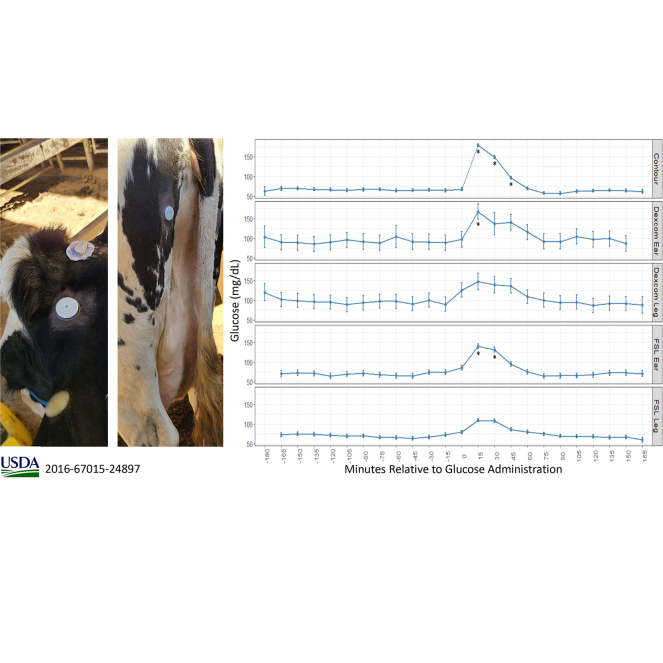
**Summary:** The purpose of this study was to determine whether interstitial glucose measurements collected by sensors designed for humans could replace blood-based glucose measurements in dairy cows. Lactating Holstein cows (n = 21) were fit with indwelling jugular catheters, as well as FreeStyle Libre (FSL; Abbott) or Dexcom G6 (DexCom Inc.) interstitial glucose monitors secured either near their ears or on their upper rear legs. Functional longevity of the sensors was greatest for those sensors secured near the ear. Blood glucose concentrations were most closely correlated with interstitial measurements from the FSL ear (r = 0.82) and the Dexcom G6 ear (r = 0.71), but accuracy was low. Both ear sensors detected an increase in glucose concentrations following a bolus dose but neither produced results exactly matching the blood glucose measurements. The results of this work indicate that both sensors can detect large changes in glucose, but neither is currently capable of replacing blood-based glucose measurements in dairy cows.

Continuous interstitial glucose sensors (commonly known as continuous glucose monitors; **CGM**) were originally developed as an alternative method of monitoring glucose in human patients with diabetes. These sensors have proven to be effective for disease management because they increase the amount of time the user spends in the targeted glucose range while also decreasing hypoglycemic and hyperglycemic conditions during both the day and night (Garg et al., 2006; Mariani et al., 2017; Didyuk et al., 2021). Glucose measurements are produced by these sensors when glucose oxidase reduces the available glucose, resulting in an electrical current that is measured (Ginsberg, 2007) and converted to a reading by each manufacturer's algorithm.. The frequency of the measurements varies between sensor devices, often ranging between 1 and 5 min. These measurements are stored in the device for download by the user and provide much more information than what would be obtained through occasional fingersticks. The user is also able to determine what direction their glucose concentration is trending, and alarms can be set to alert the wearer if their glucose concentration becomes too low or too high.

As the technology surrounding CGMs has continued to improve, there has been increasing interest in validating these sensors for use in animals. Experiments have been conducted, with some success, in horses, cats, and dogs (Reineke et al., 2010; Corradini et al., 2016; Malerba et al., 2020). To our knowledge, only limited work has been conducted in cows, using an older, implantable model of these sensors (Wiedmeyer et al., 2005). Therefore, our objective was to validate interstitial blood glucose monitors for use in dairy cattle.

This technology has the potential to be useful for both researchers and producers. Traditional methods of glucose measurement require that a blood sample be collected. Depending on how frequently samples are needed, blood is collected from an intravenous catheter or through venipuncture. With either approach, cattle may experience mild pain and stress (either as a result of venipuncture or catheter placement and the associated restraint), most of which could be avoided by using CGMs instead. In instances where frequent measurements are needed, catheter failure is an inherent risk and often requires recatheterization and loss of data. If effective, CGMs would benefit the animal by reducing pain and stress while also increasing the frequency of measurements. In research and clinical applications, these advantages represent vast improvements over the methods of glucose measurement currently in use. In addition, if these sensors could be validated for use in dairy cattle, it would allow for the development of new on-farm strategies for the detection and management of metabolic diseases, especially those prevalent during the transition period. Automated collection of glucose concentrations through CGMs and analyses of day-to-day variations for individual animals could prove useful for early detection of conditions that are detrimental to animal health and productivity (e.g., ketosis; Ruoff et al., 2017).

The primary objectives of this study were 3-fold. The first objective was to determine whether commercially available CGMs would record measurements when applied to dairy cows. Assuming glucose readings were available, we then aimed to observe the functional lifespan of the sensors and compare interstitial glucose measurements collected by the sensors with blood-based glucose values.

All husbandry and experimental procedures performed during this experiment were approved by the Virginia Tech Institutional Animal Care and Use Committee (Blacksburg).

In the first experiment, FreeStyle Libre sensors (**FSL**; Abbott) were applied with only minor modifications to the manufacturer's instructions (described below). Two locations were evaluated on 13 lactating Holstein cows from the Virginia Tech Dairy Science Complex (Blacksburg). Interstitial glucose sensors were placed directly behind the cows' polls (lateral to their ears) and beneath their pin bones on their upper rear legs. These locations were chosen because they experience less skin movement and have thinner skin than most other parts of the cow's body. Interstitial glucose sensors were adhered after shaving the area down to the skin and cleaning with an alcohol wipe. To ensure better adhesion, cyanoacrylate adhesive was added to the transmitter patch just before application (Wiedmeyer et al., 2003; Johnson et al., 2011; Hug et al., 2013). The cows utilized for this initial experiment were housed in tiestalls and were part of a larger study that included hyperinsulinemic-hypoglycemic clamps (**HHC**) and euglycemic clamps (**EC**) (necessitating placement of 4 bilateral indwelling jugular catheters). Briefly, the goal of the HHC was to maintain blood glucose at 90 ± 10% of baseline blood glucose concentrations while inducing hyperinsulinemia for 96 h (purified bovine insulin; I5500, Sigma-Aldrich Inc.; infused at a rate of 0.3 μg/kg of BW per hour). Subsequently, cows were subjected to heat stress that naturally caused them to become hyperinsulinemic and hypoglycemic. The EC was conducted during the heat stress treatment with the aim being to return cows to euglycemia (100 ± 10% of thermal neutral baseline) through infusion of 50% dextrose (Nova-Tech Inc.). The duration of this clamp was also 96 h. During the clamp periods, readings were collected from the CGMs every 4 h.

In the second experiment, 8 lactating Holstein cows from the Virginia Tech Dairy Science Complex were enrolled. On d 1, bilateral indwelling jugular catheters were placed, for a total of 2 jugular catheters per cow. Application of the FSL and Dexcom G6 sensors (DexCom Inc.) proceeded according to slightly modified manufacturers' instructions, with each cow receiving one of each sensor at each location, as previously described. FreeStyle Libre sensors were active 1 h after placement, and Dexcom sensors were active 2 h after placement.

Once all sensors were active, a 6-h sampling period began. Blood samples were collected every 15 min and glucose concentrations were measured using a handheld glucometer previously used for dairy cattle (Contour Next EZ; Ascensia Diabetes Care US Inc.). The reader for the FSL system was also capable of measuring blood glucose via testing strips, and those measurements were collected as additional information. The FSL sensors were scanned with the respective readers each time a blood sample was collected. Dexcom data were uploaded to a computer at the end of the 6-h sampling period, and times recorded were paired with those of blood glucose measurements.

To test the lag time between changes in blood glucose and changes in interstitial glucose as detected by the sensors, a glucose challenge was administered approximately halfway through the sampling period. At hour 3, cows were given an intravenous bolus of 50% dextrose (0.3 g/kg of BW; Durvet Inc.), which was infused through the catheter that was not being used for blood sample collection. Measurements were taken every 15 min throughout the sampling period.

At the end of the 6 h, the jugular catheters were removed, and cows returned to their freestalls with all working sensors still secured. Sensors were checked once a day for the following week until sensors were no longer providing glucose readings (i.e., produced an error code or read “lo” at multiple collections where blood glucose was >40 mg/dL) or became unattached.

The resultant data sets included some or all combinations of the following: time-paired blood glucose readings from Contour blood glucose readers, FSL blood glucose readers, FSL CGMs placed on the hind leg, FSL CGMs placed near the ear, Dexcom CGMs placed on the hind leg, and Dexcom CGMs placed near the ear. For analyses, the CGM data points were paired with blood glucose reader data points from 15 min earlier, in accordance with findings from research conducted in horses (Johnson et al., 2011). This approach was confirmed in the current study using a subset of samples that had been collected every 5 min from the time of dextrose administration to 30 min post-administration (data not shown). Paired correlational analyses of all possible variables were performed using the cor.test function in the stats package, version 4.0.3 in R (https://www.R-project.org/). By chance (due to nonfunctional or failed sensors), simultaneous paired measurements from some combinations of CGMs were not available. Likewise, the Dexcom CGMs were not utilized alongside FSL blood readings. For these reasons, we were unable to make comparisons between the FSL leg sensors and either Dexcom sensor or between the FSL blood readings and either Dexcom sensor. Pearson correlation coefficients were considered significant if *P* < 0.05. Based on the findings of the regression analyses and on the presence of highly influential points in the comparison of the Dexcom sensors with the blood glucose readings, we determined that further analyses would be relevant only for the comparisons of the FSL ear sensor with the 2 blood glucose readers.

The absolute relative error (**ARE**) for each FSL ear CGM data point with the respective data point for each blood glucose monitor was calculated using the formula below:

$$ARE=\frac{CGM\;reading-\;Blood\;glucose\;reading}{Blood\;glucose\;reading}\times 100.$$

Because readings with an ARE <20% are not considered to differ significantly from the original reading (Clarke et al., 1987; Ginsberg, 2009), the percentage of points with an ARE <20% was determined. When all data were included, none of the CGMs produced a satisfactory percentage of points below the 20% threshold, so the data points from the glucose tolerance test were removed and the calculation was performed again.

As a follow-up, we performed separate simple linear regression analyses for each blood glucose reader using the CGM reading as the only regressor and measurements from one blood glucose reader as the response variable. The goal of this analysis was to determine whether a predictive equation could be created to produce accurate blood glucose readings using the CGM reading. The linear equation was determined to be efficient in producing accurate readings if the R^2^ value was greater than 0.90. To confirm these results, a new ARE was calculated using the predicted blood values for each blood glucose reader from the linear equation in place of the CGM reading, and the percentage of data points with an ARE <20% was again determined.

For the second experiment, glucose concentrations over time were analyzed using the MIXED procedures of SAS (SAS Institute Inc.). Cow was included in the model as the random variable. For each analysis, 8 covariance structures were tested and the most appropriate was selected based on Akaike's information criterion, Akaike's information criterion with correction, and Bayesian information criterion values. Results are reported as least squares means ± standard errors of the means. Means were separated using the Tukey procedure of SAS. Statistical significance was declared at *P* < 0.05 and a tendency for a difference at 0.05 < *P* < 0.15.

The first experiment addressed the first objective of determining whether readings could be obtained from CGMs applied to dairy cows. The sensors did indeed report measurements, thereby justifying further investigation. Although not all aspects of the larger experiment were ideal for CGM assessment, the frequent monitoring necessitated by the HHC and EC was advantageous for determination of sensor functional lifespan (Table 1). During these clamps, observations were collected every 4 h, whereas sensors were checked only once daily for the cows in the second experiment. The longevity of the FSL sensors was affected by site of application (ear vs. leg). Sensors near the ears lasted over 3 times longer (*P* < 0.001) than sensors on the legs. Interestingly, the type of clamp (HHC vs. EC) also affected longevity, with sensors applied during the EC lasting over 3 times longer than those applied during the HHC.

**Table 1:** Functional lifespan (hours:minutes) of FreeStyle Libre (Abbott) sensors during the first experiment

Item	n	LSM	SE	*P*-value
By period^1^				
HHC	13	14:52	5:53	<0.01
EC	10	49:38	6:37	
By location^2^				
Ear	13	48:37	5:53	<0.01
Leg	10	15:52	6:37	

1 Sensors were placed at the beginning of hyperinsulinemic-hypoglycemic clamps (HHC) and euglycemic clamps (EC).

2 Sensors were placed near the ear or on the rear upper leg.

During the second experiment, data were gathered from 8 FSL ear sensors and 8 FSL leg sensors. One cow's FSL ear and leg sensors were removed from the statistical analyses because the sensors only worked for a short time. Although 16 attempts were made, only 4 Dexcom ear sensors and 2 leg sensors were applied, and only 2 ear sensors and 1 leg sensor produced enough readings for analysis (n = 2 cows). As a result, functional duration in the freestall environment could only be evaluated for the FSL sensors. The longest-lasting sensor provided measurements for 5 d and it was one affixed near the ear. Sensors attached to the rear leg functioned for a maximum of 2 d. Overall, the majority of the FSL sensors remained functional for at least 1 d in the freestall pens.

Glucose concentrations as measured by the Contour, Dexcom ear location, and FSL ear location during the 6-h sampling period differed over time (*P* < 0.01 for each; Figure 1). Glucose concentrations measured by the FSL leg location tended to differ over time (*P* = 0.11). The dextrose bolus administered during the glucose challenge elevated blood glucose measurements (measured with the Contour) at 15, 30, and 45 min post-bolus. None of the other devices detected differences in glucose concentrations at all of the same time points. The FSL ear analyses were most similar, where glucose concentrations differed from baseline at the 15- and 30-min time points.

**Figure 1:**
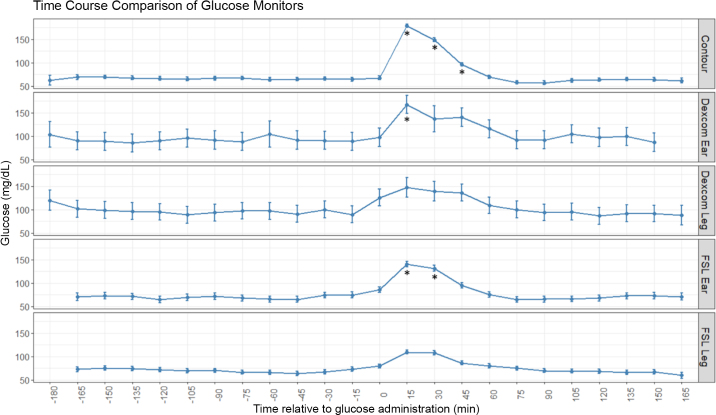
Glucose concentrations as reported by the Contour Next EZ (Contour; Ascensia Diabetes Care US Inc.), Dexcom G6 (DexCom Inc.), and FreeStyle Libre (FSL; Abbott). The Dexcom and FSL continuous glucose monitors were applied either behind the poll near the ear or on the upper rear leg. At 0 min, a dextrose bolus was administered (0.3 g/kg of BW); glucose concentrations as measured by the Contour, Dexcom ear location, and FSL ear location differed over time (*P* < 0.01 for each). Asterisks indicate measurements that were greater than baseline values (*P* < 0.05).

Blood glucose values from Contour measurements were most highly correlated with the FSL ear sensor (Table 2) and the Dexcom ear sensor and were analyzed further. Although significant, the correlations between blood glucose and the sensors applied to the rear legs were low (Table 2). The correlation between the FSL blood glucometer and the FSL leg sensor was also weak (Table 2). The relationship between the FSL blood glucometer and the FSL ear sensor was intermediate (Table 2) and was analyzed further.

**Table 2:** Correlations (r) between FreeStyle Libre (FSL; Abbott) continuous interstitial glucose monitors (CGMs), Dexcom CGMs (DexCom Inc.), and blood glucose readings from Contour Next EZ (Contour; Ascensia Diabetes Care US Inc.) and FSL blood glucometers

x		y	r	*P*-value
Contour		FSL Ear	0.819	<0.001
Contour		FSL Leg	0.688	<0.001
Contour		Dexcom Ear	0.713	<0.001
Contour		Dexcom Leg	0.413	<0.001
Contour		FSL Blood	0.748	<0.001
FSL Ear		FSL Leg	0.781	<0.001
FSL Ear		Dexcom Ear	0.823	<0.001
FSL Ear		Dexcom Leg	0.603	<0.001
FSL Ear		FSL Blood	0.748	<0.001
Dexcom Ear		Dexcom Leg	0.470	<0.001
Dexcom Ear		FSL Blood	0.838	<0.001
FSL Leg		FSL Blood	0.564	<0.001
FSL Leg		Dexcom Ear	0.527	<0.001
FSL Leg		Dexcom Leg	0.512	<0.001

Equations were fit to the scatterplots for the FSL ear sensor compared with the Contour (y = −13.55 + 1.115x; R^2^ = 0.67) and the FSL blood glucometers (y = −4.704 + 0.8507x; R^2^ = 0.56). An equation was also generated for the scatterplot comparing the Dexcom ear to the Contour (y = 5.899 + 0.6812x; R^2^ = 0.51). All resulting R^2^ values were low.

When the sensors were compared with blood readings and assessed for accuracy (<20% ARE; Clarke et al., 1987; Reineke et al., 2010), only 47.0% of the points were within range for the FSL ear compared with the Contour. The number of measurements considered accurate increased to 60.7% when data from the glucose tolerance test was removed. The accuracy for the FSL ear sensors compared with the FSL blood glucometer was 22.4% and decreased to 17.4% when the glucose tolerance test data were removed.

Although the overarching hypothesis of this work was that CGMs developed for use in humans could be validated for use in dairy cattle, the first objective was simply to determine whether these commercially available CGMs would record measurements following application to dairy cows. Holstein dairy cows are dissimilar to humans in several ways that could have prevented the CGMs from collecting any measurements at all. First is the difference in average circulating glucose concentrations. Circulating glucose concentrations in humans are generally 80 to 140 mg/dL, whereas blood glucose concentrations in dairy cattle are considerably lower (42–75 mg/dL). Because the CGMs were developed for use in humans, their detection range is not ideal for dairy cows. The minimum detection level is 40 mg/dL for the FSL and the Dexcom. While this is sufficient under most circumstances, it is problematic for cows in hypoglycemic conditions.

Success of the first objective was dependent upon identification of an application site with comparatively thin skin and where there was minimal movement of the skin over the underlying tissue. In addition to these criteria, the site of application had to be a location where the cow could not easily dislodge the sensor. These criteria severely limited candidate sites for application. Furthermore, these sensors were designed for use in humans with an average skin thickness of 2.29 mm for the abdomen and 2.00 mm for the upper arm (locations used for sensor placement; Sim et al., 2014), whereas skin thickness in cattle ranges from 2.49 to 5.37 mm (Hamid et al., 2000). To further complicate matters, the sensors should also be placed in adipose tissue and not too close to muscle. If the sensors are too close to muscle, the glucose concentrations will read inaccurately low (Hug et al., 2013; Corradini et al., 2016). Proximity to muscle is likely why the sensors applied to the legs generated readings that were poorly correlated with blood values. The correlations were low for both the FSL and Dexcom leg sensors, regardless of which handheld blood glucometer was used for comparison. In general, the application site behind the poll lateral to the ear was highly correlated with blood measurements for both sensor types. It is possible, however, that other sites of application would yield more reliable measurements than those tested here. Wiedmeyer and coworkers (2005) tested a different company's CGM on 5 dairy cows and found a strong correlation between interstitial glucose and blood glucose when the sensor was placed on the lateral neck. It is important to note that this CGM was an older model where the electrode was inserted into the tissue with a stylet. Regardless of sensor type, location influences the accuracy of the sensors and, therefore, alternative locations may prove to be more accurate than the leg and behind the poll.

Application of the FSL was easy, with only some of the animals momentarily bothered by the clicking of the applicator near their head during ear application. When the sensors were removed, the skin beneath them did not exhibit any adverse reactions. One cow experienced slight swelling from an FSL near her ear and she also bled briefly after the initial application. That cow's FSL ear sensor was one of the sensors that was removed from analyses for not working long enough to produce sufficient data. It seems likely that a small superficial blood vessel was ruptured at the site of application. None of the sensors from either experiment produced serious adverse reactions. Unfortunately, the Dexcom CGMs presented several challenges to use and evaluate in cows. The vast majority of the sensors did not deploy properly from the applicator, and in fact, became permanently irremovable from the applicator. Investigation of this issue revealed that this is a common problem even when applied to humans. This issue ultimately resulted in 10 sensors failing to deploy, decreasing the amount of data we were able to collect and making it difficult to draw any meaningful conclusions.

The second objective of this work was to observe the functional lifespan of the sensors. Unfortunately, the small number of successful Dexcom applications prevented statistical analysis of its functional longevity. For the FSL CGMs, the most informative data were gathered during the HHC and EC as a result of the frequent measurements. Although the functional lifespan of the FSL applied to dairy cows was considerably less than the advertised longevity for humans (14 d), it was sufficient to be useful for many applications, research and otherwise. In regards to location of the sensor, the FSL CGMs applied near the ear lasted more than 48 h. The location-based difference in functional lifespan is likely the result of better adherence to the location criteria. Unexpectedly, the HHC sensors ceased functioning far sooner than the sensors used during the EC. Although the HHC induced hypoglycemia, blood glucose seldom fell below the FSL minimum detection limit of 40 mg/dL. Thus, it is unclear why the HHC sensors failed sooner than EC sensors. In support of these findings, however, experiments conducted in dogs and humans demonstrated that during hypoglycemic events, FSL sensors decreased in accuracy compared with euglycemic and hyperglycemic conditions (Corradini et al., 2016; Fokkert et al., 2017).

The third and final objective of this work was to compare interstitial glucose measurements collected by the sensors to blood-based glucose values. Correlations between the CGMs applied near the ear and blood values (as measured with the Contour) were >0.70, and some locations of both sensor types detected the dextrose bolus administered during the glucose challenge. Although these aspects of the analyses were promising, according to our results, neither the FSL nor Dexcom CGMs can be used at this time to accurately represent circulating glucose in lactating dairy cattle. Accuracy of the FSL sensors was 47%, when ideally that value should approach or exceed 90%. The accuracy of the Dexcom could not be calculated because so few of them successfully deployed from their applicators. The limited results gathered from Dexcom sensors imply that they would warrant further investigation if the deployment problem was solved. Accuracy of both sensors might also be improved through development of cow-specific algorithms for each sensor. The FSL and Dexcom come calibrated from the manufacturer, and as a result, use an algorithm specifically designed to convert interstitial glucose readings of humans to useable glucose concentrations. It is unlikely that the optimal algorithm for humans would also be optimal for generating glucose concentrations for cows.

Unfortunately, there is no easy solution to address the minimum detection limit of the CGMs. While a minimum detection limit of 40 mg/dL is appropriate for humans, average glucose concentrations in dairy cows are lower and approach the minimum detection limit of the CGMs. This could explain why the accuracy for the FSL ear sensor compared with the FSL blood glucometer decreased when the data points following the glucose bolus were excluded from the analysis. The initial reasoning for excluding those data points was that the interstitial glucose concentration seemed to stay elevated for a longer duration, even after the blood glucose concentrations had declined to baseline levels. However, excluding those points reduced the accuracy of the FSL-ear/FSL-blood comparison. This indicates that many of the accurate points were obtained while the cows were hyperglycemic. Therefore, increased accuracy seemed to occur at higher interstitial glucose concentrations.

Aspects of interstitial glucose monitors could make them a useful tool for monitoring a dairy cow's glucose concentrations if the existing challenges could be resolved. The currently available devices are capable of detecting sizeable changes in blood glucose, such as might be observed during a glucose tolerance test. Unfortunately, overall, they appear to be poor indicators of blood glucose concentrations in dairy cows. Obstacles including issues with application, calculation of algorithms, and inappropriate minimum detection limits will need to be further explored and resolved. Overcoming these obstacles will enable CGMs to be employed as a useful tool for researchers, clinicians, and producers needing to closely monitor glucose concentrations in dairy cows.
